# Coping and adaptation process during puerperium

**Published:** 2012-06-30

**Authors:** Angélica María Ospina Romero, Lucy Muñoz de Rodríguez, Carmen Helena Ruiz de Cárdenas

**Affiliations:** a Faculty of Nursing and Rehabilitation, Universidad de La Sabana. E-mail: angelica.ospina@unisabana.edu.co; b Universidad de La Sabana, , Faculty of Nursing and Rehabilitation, Universidad Nacional de Colombia, Faculty of Nursing, Faculty of Nursing and Rehabilitation Universidad de La Sabana Faculty of Nursing Universidad Nacional de Colombia. E-mail: lucymdero@yahoo.com

**Keywords:** nursing, adaptation, postpartum, pregnancy, family

## Abstract

**Introduction:**

The puerperium is a stage that produces changes and adaptations in women, couples and family. Effective coping, during this stage, depends on the relationship between the demands of stressful or difficult situations and the recourses that the puerperal individual has. Roy (2004), in her Middle Range Theory about the Coping and Adaptation Processing, defines Coping as the ''behavioral and cognitive efforts that a person makes to meet the environment demands''. For the puerperal individual, the correct coping is necessary to maintain her physical and mental well being, especially against situations that can be stressful like breastfeeding and return to work. According to Lazarus and Folkman (1986), a resource for coping is to have someone who receives emotional support, informative and / or tangible.

**Objective:**

To review the issue of women coping and adaptation during the puerperium stage and the strategies that enhance this adaptation.

**Methods:**

search and selection of database articles: Cochrane, Medline, Ovid, ProQuest, Scielo, and Blackwell Synergy. Other sources: unpublished documents by Roy, published books on Roy´s Model, Websites from of international health organizations.

**Results:**

the need to recognize the puerperium as a stage that requires comprehensive care is evident, where nurses must be protagonist with the care offered to women and their families, considering the specific demands of this situation and recourses that promote effective coping and the family, education and health services.

## Abstract (es)

**Introducción:**

el puerperio es una etapa que produce cambios y adaptaciones en la mujer, pareja y familia. El afrontamiento eficaz, durante esta etapa, depende de las relaciones entre las demandas de las situaciones estresantes o difíciles y los recursos que la puérpera posea. Roy (2004), en su Teoría de mediano rango, sobre el Proceso de Afrontamiento y Adaptación, define el Afrontamiento como ''los esfuerzos comportamentales y cognitivos que realiza la persona para atender las demandas del ambiente''. En la puérpera el afrontamiento adecuado es necesario para mantener el bienestar físico y mental, en especial frente a situaciones que pueden ser estresantes, como la lactancia y el regreso al trabajo. De acuerdo con Lazarus y Folkman (1986), un recurso de afrontamiento es tener alguien de quién recibir apoyo emocional, informativo y/o tangible.

**Objetivo:**

revisar el tema sobre el afrontamiento y adaptación de la mujer, durante la etapa del puerperio y las estrategias que favorecen esta adaptación.

**Métodos:**

Se recopilaron artículos de investigación y sistematización relacionados con el tema en las bases de datos de Cochrane, Medline, Ovid, ProQuest, Scielo, Blackwell Synergy. Otras fuentes: documentos inéditos de Roy, libros publicados sobre el modelo de Roy. Sitios web de organizaciones de salud internacionales.

**Resultados:**

se evidencia la necesidad de reconocer el puerperio como una etapa que requiere atención integral, donde enfermería debe ser protagónica con el cuidado brindado a la mujer y su familia, considerando las demandas específicas ante esta situación y los recursos que favorezcan el afrontamiento eficaz como, la familia, la educación y los servicios de salud.

## Introduccion

This work contains the review and critical analysis of the issue of the puerperium as a process of coping and adaptation, considering that this stage involves physiological, psychological, socio-family, and cultural changes and adaptations for women and their families^1^ ^-^ ^3^.

Physiologically, the puerperium is considered the period of time transpired from the expulsion of the placenta until the return of the woman's reproductive organ to its state prior to gestation^4^. From the psychological and social points of view, it is a period of re-adaptation and adjustment for the whole family; the woman may end up feeling relegated, given that the attention is centered on the newborn and she may even have to modify her role to dedicate herself to caring for the newborn, in spite of the physical discomfort caused by giving birth^5^.

From the cultural vantage point, the puerperium is related with the 'diet' considered within the 40 days postpartum. Care for the woman and her child centers around feeding, recovering the figure, eliminating cold flashes from the body, reinitiating sexual activity, restricting physical activity, breastfeeding, and preventing disease in the newborn, with talismans or traditional practices^6^.

Considering puerperium from an integrality stand point and as a process requiring attention from healthcare services to favor the coping and adaptation process in women, the revision of this theme has been motivated, within the framework of a revision that seeks to offer guidelines for interventions that favor coping and adaptation of women during this stage.

This revision of the theme reveals research and experiences on the puerperium, related to aspects that can impact upon the coping and adaptation process and which must be kept in mind for the care offered to women and their families. The first part, a comprehensive look at the puerperium reviews two aspects: the puerperium from the different modes of adaptation according to Roy's model; the physiological, the self-concept, the role, the interdependence; the second aspect is the coping and adaptation process in the puerperium. The second part poses problems of adaptation and support recourses, among those the family is found, education as an element of care and its relation with the coping and adaptation process, and, finally, maternal perinatal healthcare norms and the role of nursing during the puerperium.

## 1. A comprehensive look at puerperium

During the puerperium, external and internal stimuli trigger in women the subsystems of regulating and cognitive coping, which generate behaviors or responses evidenced through four manners: the physiological, the self-concept, the role, and the interdependence. The regulating subsystem is mediated by the Autonomic Nervous System and the Endocrine System and its responses are reflected in the physiological mode. The cognitive subsystem, on its part, is frame worked in the conscience, and is related to cognitive and emotional processes, which are founded on experience and education^7^.

### 1.1 adaptation modes

#### The physiological.

This mode of adaptation becomes evident through the regulating mechanism. Changes occur inside the organism because of effects of gestation and childbirth; the most significant are related to uterine involution, recovery of vaginal tone and of the external genitalia, return of the tone of the abdominal wall, and stabilization of the endocrine, cardiovascular, and urinary functions. The period of physical adjustment can last between the fourth and sixth week postpartum^8^. The sudden absence, during postpartum, of steroid hormones produced during gestation has a direct effect on the woman's frame of mind, related to postpartum depression^9^ ^,^ ^10^ ^.^ Some functions, like the cardiovascular, can require months to become stabilized^11^.

#### The self-concept.

This mode is related to the adoption of behaviors, which may or may not be healthy^12^. It has been found that discomfort related to postpartum is perceived as normal. In a survey conducted in the United States with puerperium women, it was concluded that ''many of the new health problems persisted, at least, during six months after childbearing, but most women never said anything to the healthcare provider''^13^.

#### The role function.

This mode of adaptation of women during puerperium is related especially to the different roles they must perform, that is, that of maternity, spouse, and worker. Emmanuel et al., (2008) concluded that social support is the most important factor for the development of the maternal role. Successful adaptation to this function provides confidence and satisfaction to the mother in her capacity to care for her baby^14^. Nichols and Roux (2004) described maternal perception and returning to work during postpartum as a negative experience, with more challenges than expected. Preparing to again assume the former role was important, but unpleasant. Some positive aspects are highlighted, like satisfaction for their professional role and the maternal role^15^.

Regarding the role with their spouse and with their sexual life, this is generally diminished. Aspects like vaginal bleeding, perineal discomfort, hemorrhoids, breast pain, and decreased vaginal lubrication associated to breastfeeding, aggravated by the fatigue of restless nights, contribute to diminished motivation for sexual activity. Other factors like fear of waking the baby, the sense of reduced attraction, change of bodily image, or changes in frame of mind also influence. Most do not speak of the issue with their partners or with healthcare professionals, who seem uncomfortable dealing with the matter^16^. Addressing sexuality issues in nursing can contribute to the woman's health, as long as the nurse is adequately prepared for such and manages to establish empathy. These problems can be solved by providing clear information, within a comfortable and reliable setting. Some disorders, however, will require bio-psychosocial support from other disciplines.

#### Interdependence.

As an adaptation mode, it involves the affective and social relationships women establish with significant individuals and with support systems. In it, socio-cultural aspects surrounding the gestating woman become important. In an investigation conducted by Argote *et al*., (2004)^17^, puerperium is considered the transition of status from woman to mother, differentiating the following stages:

#### * The separation: 

the woman is physically separated; she has restrictions for eating, bathing, going to the street, and for engaging in sexual intercourse. Her social relationships are reduced, in great part, to family gatherings.

#### * The liminality:

appears on the 40th day, when the social restrictions increase, she is accompanied by a female family member, they perform an incense rite and the bath to eliminate the bad gathered during the quarantine or postpartum period. 

#### * Entrance to a new social state:

occurs after the 40th day, when the woman can progressively assume daily activities^17^. 

Puerperium is also considered a stage of transition. In this regard, the Medium-range Theory of transitions, described by Schumaker and Meleis, has been used to identify the elements that characterize the transitions of gestation and postpartum. During this stage, women undergo changes in their external and internal world and in how they perceive them. If women know what can happen during the postpartum, the anxiety associated diminishes and their coping and adaptation capacity improves^18^.

Alves (2007) inquired on the perceptions of the transition to the maternal role of primiparous puerperium women, finding that it is possible to understand the experience of women on their way to the maternal role: their feelings, accomplishments, difficulties, changes imposed by the baby's arrival^19^. Zagonel *et al*., (2003) concluded that nursing care has as a goal to offer coping and adaptation strategies during the transition to maternity, through professional support networks working on the perceptions and feelings from the transition and the difficulties this stage produces^20^.

The adaptation modes described permit recognizing that these are present in women during puerperium, but healthcare personnel emphasizes specifically on the physiological, leaving aside the other adaptation modes, such as, the self-concept, domain of the role, and interdependence, which could limit comprehensively approaching women during this stage, insofar as opportune diagnosis and management.

### 1.2 Coping and adaptation during puerperium

Some investigations on coping have centered on establishing the relationship between the strategies used under stressful situations and the health status; it was demonstrated that, as strategies became effective, health problems were lessened. Among the recourses indicated during puerperium, there are self-esteem, prior experiences, permanent learning, knowledge, beliefs, and the organization to return to work^21^. This is evidenced by the cognitive subsystem that contemplates perception, emotion, learning, and judgment.

In perception, prior experiences become important in the coping process. Mercer references that, in relation to their children, women reconstructed the maternal model and those who remembered having been accepted by their mothers, who currently had a balanced relationship with them, showed to be more sensitive in child rearing; on the contrary, rejection during their childhood was a predictor of depressive symptoms^22^.

Emotion is manifested through defense mechanisms to relieve anxiety, via responses like crying, despair, or angst; these responses produce effects like tachycardia, tachypnea, and even, depression. It can also be manifested with joy, satisfaction, desire of unconditional dedication in caring for the child, strengthening the maternal bond^5^.

Learning involves imitation, reinforcement, and insight and implies the domain of necessary skills to perform the maternal role^23^; here, beliefs intervene, which influence hoe postpartum is confronted.

Judgment comprises decision making and problem solving, becoming evident during postpartum, where women adjust to new challenges and pose solutions, which involve their beliefs, experiences, values, principles, feelings, and education. In research conducted by Knaak^24^, women manifested that among the main recourses used in the process of adjustment to maternity, there are prioritizing their self-care, controlling stressful situations, having sufficient help, and having real expectations.

As can be noted, considering the cognitive subsystem is fundamental, given that it is present in the woman's decision making, it is part of her everyday life, and influences directly on her self-care and the care she offers her child. For example, not washing her hair during puerperium or placing a bracelet on the wrist of the newborn to avoid the evil eye.

## 2. Problems of adaptation and support recourses

**Figure 1:**
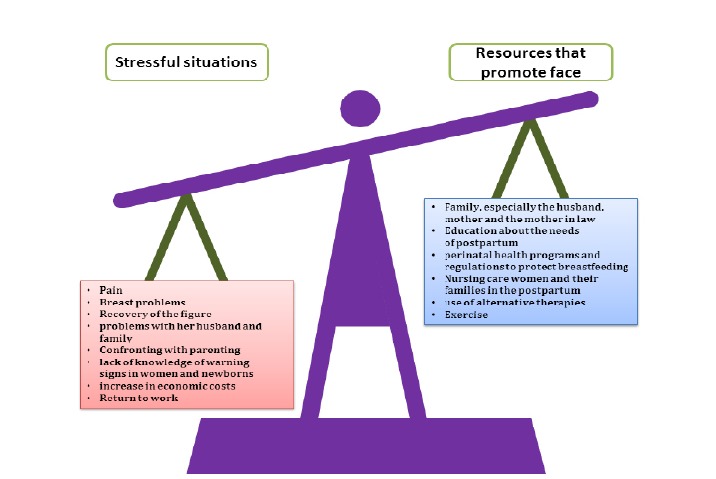
Efficient coping during puerperium Balance Between

When women fail to maintain the equilibrium between the demands of the situation and the recourses puerperium women have to confront such situation, they may enter cognitive, emotional, and motivational deficit, which will lead them to non-adaptive conducts like depression. On the contrary, women who manage to use these recourses adapt more easily to stressful situations (Fig.1).

Hung (2004) determined the effects of postpartum stress, depression, and social support on the health of puerperium women, finding that stress is related more with life events than with the tension of the adjustment period. Women who were breastfeeding had a high level of social support against those using infant formula; those with high educational levels, full-time work, higher income, and social support had lower levels of depression^25^.

Poof (2008) investigated on the risk factors associated to postpartum depression and found that most are of family type, with the interactions among the family, including the spouse, having the greatest repercussion on the woman's affectivity^26^. It was also observed that in cultures where psycho-social support is established during pregnancy, childbirth, and postpartum, the incidence of postpartum depression is low^27^.

Postpartum depression has a direct relation on maternal adaptation and the care for the child. Barr (2008) concluded on women with this diagnosis that their adaptation to the social role and their competency in caring for the children are delayed, doing this work in mechanical manner. It was also concluded that the education received in prior phases can facilitate an adequate adaptation for them^28^. Barlow and Coren (2008) analyzed follow-up studies that suggest that the psycho-social health of the mothers can have a considerable effect on the mother-child relationship, and mental-health problems, like postpartum depression, give way to cognitive and emotional deficits in the child^29^.

Although postpartum depression is an important adaptation problem, it is in everyday life where difficult situations exist, generating conflict in women and their families. Escuriet and Martínez (2004) investigated on the health problems and the motives for concern perceived by puerperium women prior to hospital discharge. They reported that the most frequent were pain and problems with the breasts; the psycho-emotional aspects that concerned most the primiparous women were the perception on their capacity to care and their loss of autonomy^30^.

Martin (1995) analyzed, through a descriptive study with Roy's model, the most common problems during postpartum in the first two to eight weeks. The results reported: sleeplessness, feeling tired, stressed, discouraged, and lonely, increased household work, loss of income, concern with the personal appearance, unable to concentrate, concern regarding how to face the role of mother and change in sexual desire^31^.

The woman's perception of her bodily image can also be altered. Generally, there is discomfort with the physical appearance, due to the weight gain during gestation and the sequelae on the facial skin and the abdomen, which may even contribute to diminished self-esteem. Add to this the habitual beliefs during postpartum, when women are overfed and remain at rest, leading them to rapid weight gain. Research conducted to explore the experiences of women with their bodies and frame of mind during gestation and postpartum reported that, during pregnancy, there are aspectshat help them to positively face the bodily changes; however, during postpartum, lack of satisfaction is manifested with this image. They state they no longer have an excuse to seem pregnant and tyearn to return to normality.

The implications of these findings show the need to offer education on the normal changes during this time and the aspects related with the bodily image^32^.

Physical exercise acts as a stimulus to manage the bodily image and promote effective responses. Generally, this practice starts as of gestation and is related with fetal wellbeing; during postpartum, instead, with weight loss. Blum *et al*., (2004) examined changes in physical activity and maternal wellbeing during postpartum. They concluded that sports and exercise habits in women become predictors of maternal wellbeing. Support from the family and spouse was an influencing factor in maintaining exercise habits^33^. Likewise, women who engage in vigorous exercise during postpartum show better results in adaptation, with more possibilities of participating in socialization activities, hobbies, and entertainment, compared to those who do not perform said type of exercise^34^.

The use of alternative therapies can also support the physical and mental state of puerperium women. Imura *et al*.,(2006) conducted an investigation with two groups of women who were in the second day of postpartum; one group received a massage with aromatherapy and the other was offered conventional attention, revealing that the massage with aromatherapy is effective to improve the physical and mental state of the mother and to facilitate the mother-child interaction^35^.

### 2.1 The family as a coping strategy

The arrival of a new member into the home produces an evolutionary crisis in the family, which requires special care, given that its vulnerability and transcendence makes it a generator of health or illness. According to Roy's Nursing Model, the family is a factor that intervenes in the health of the individuals, and it can be evaluated in the four adaptive modes - physiological, group identity, role domain, and interdependence^23^.

The physiological mode evaluates the physical necessities of the family; it is directly related to the other adaptive modes and permits seeing the family group in holistic terms. Group identity (equivalent to the self-concept for the individual) reflects how individuals see themselves in the group, based on the feedback from the environment on their interpersonal relationships, goals, and values; the role may be evaluated through the communication among its members; the integration of the different roles determines their level of adaptation; interdependence evaluates their interaction with society and the support systems^23^.

Frequently, the family constitutes a stimulus that impacts upon the behavior of its members. An example of this process is noted when the mother has an illness (or problem of adaptation) during postpartum and, hence, feels incapable of performing her role. The main stimuli for the development of this behavior are the family's poor health practices. Also, her inability to perform her role diminishes the group's coping level^36^. On the contrary, Wanna (2004) found that, in Thailand, to return to work, family help with caring for the child had a positive effect in primiparous women^37^. It is thus that the family learns to identify the recourses that are useful to face stressful situations and from there accomplish equilibrium within the family dynamics.

### 2.2 Education and coping

Education implies a personal, free, voluntary process that helps to improve the individual who assumes it and which enables that person to act as a social being^38^. It provides for each person and his or her life project the stimuli and means necessary to reach desirable attitudes and habits for social and personal improvement^39^. According to Roy's model, for education to adequately promote the capacity for coping and adaptation, we must bear in mind the individual as a holistic being, placed within a social context and a changing environment^40^.

Gestation and puerperium are privileged periods for education. It would be ideal for education to surge from real necessities of gestating and puerperium women, but the current scenario is different. Not all women receive education, nor do healthcare providers have clear guidelines; although, healthcare norms require for education to be offered individually and colectively^41^. Consequently, each healthcare institution develops the educational sessions in different manner. In research with women and educators who took the prenatal courses, Renkert and Nutbeam (2001) analyzed the opportunities to improve maternal health through antenatal education. Both groups indicated time as a limiting factor. Furthermore, there is influence from anxiety and curiosity for knowing about pregnancy and childbirth. Primiparous mothers revealed that they would have wanted more information about postpartum in the antenatal classes and that these had not been of much help^42^. Hence, is current prenatal education sufficient to maintain and increase levels of coping and adaptation during puerperium? Or does it merely center on improving knowledge?

Individual education has revealed to promote higher levels of knowledge. A study by Calderón *et al*., (2008) measured the impact of a personalized educational intervention on the level of knowledge of adolescents on self-care practices during puerperium. The result was that the intervention significantly influenced on the level of knowledge of puerperium women^43^.

Dyson *et al*., (2008) conducted a meta-analysis to evaluate the effectiveness of interventions to promote the practice of women breastfeeding their newborn infants; they found that the different forms of education on maternal breastfeeding resulted efficient to increase rates of initiation of this practice. The content of the sessions was based on the needs and interests of the participants and on the preparation of the medical and nursing professionals in charge of interacting with the mother-child couples^44^.

Research found on education for maternity reflects the need for conclusive studies that investigate its effects on women and their families. Gagnon and Sandall (2008), in a systematic review conducted to evaluate the effects of education conclude that, although in many countries women and their partners are habitually referred to education programs, its benefits continue being uncertain. The widespread popularity of prenatal classes ratifies the desires of many parents (particularly first-time parents) to receive this education^45^. All this is a reference that must be kept in mind. However, guides are required, based on evidence, that orient education for puerperium women since the prenatal stage. Additionally, it is necessary to establish a maternal and neo-natal care system extended to the family, defined by Ruiz (2002) as comprehensive health-care of mothers and their newborn children based on knowledge of biological, emotional, socioeconomic, cultural, and spiritual needs^46^.

### 2.3 Norms in maternal-perinatal health

International and national approaches and regulations recognize the importance of maternity, the promotion of healthcare during this stage, disease prevention, and reduction of maternal and perinatal mortality, but there is a great diversity of women's necessities during puerperium, which are unknown by the healthcare services responsible for healthcare during this stage.

Maternal health is one of the most important determiners in community development. The Colombian Ministry of Social Protection considers that women's health, especially safe maternity and the conditions for the exercise of human rights associated to such, like the right to life, directly reflects the level of the nation's development and the degree of reduction of economic and social inequities^47^. The maternity event encompasses a high percentage of the population. Currently, in Colombia 4% of women are in gestation^48^. However, as revealed by the WHO, for 2008 there were 358,000 maternal deaths worldwide, a figure that has decreased in relation to prior years, but still remains high^49^. In Colombia, during 2008, 503 maternal deaths were registered, generated - in part - by access barriers to healthcare services and the quality of public healthcare attention^50^. The main direct cause of maternal death in Colombia is gestational hypertension^51^, which is an avoidable cause if comprehensive care is offered not only to the woman, but also to the members of her family to act as care agents.

Among the Colombian policies and guidelines that promote healthcare of women during gestation and puerperium, there are the Political Constitution; Legislation 100 of 1993, which privileges infant and maternal care; Resolution 00412 of 2000, which are the guides and technical norms of Sexual and Reproductive Healthcare; Agreement 244 by the Ministry of Social Protection, which prioritizes the entry of gestating women into the Subsidized Regime, with coverage for prenatal care, partum, and puerperium, among others^52^.

Although there are current national and international regulations, organizations like the Chilean Ministry of Health (2007) poses that, currently, there is insufficient concern regarding the needs of women during postpartum, or real awareness of their vulnerability. Added to this is that the concept of postpartum as a prolonged period is new in the healthcare culture. Furthermore, its duration is quite variable, which hinders programming its care. Also, postpartum is not recognized as an opportunity to positively influence childrearing, as parental skills are increased. There is no doubt that this is a period in which women are in different conditions to those found during other stages of their lives^53^.

### 2.4 Role of the nursing professional

Prenatal and postnatal care tends to become routine and it may be taken for granted that things are being done correctly, without their having been evaluated. Because of this, the Pan American Health Organization emphasizes that nursing care for women during gestation, partum, or puerperium requires nursing practices based on evidence to propose, from the very knowledge of the discipline, the revision of healthcare practices^41^.

Penna *et al*., (2006) researched on nursing consultation during puerperium and the experiences of women, concluding that family participation is important, as well as accompaniment, really addressing the emotions, and the rescue of the woman's autonomy^54^. But, how much are nurses concerned with caring for the real needs of women, their families, and their newborn infants?

The experiences of the parents regarding hospital and home nursing care during puerperium permit recognizing their advantages and limitations as educational settings. Two categories surge in investigations conducted on the social representations of women about the nursing care received during puerperium, and home education. Lack of satisfaction with the hospital represented as a place of abandonment, facilitated by the short hospital stay. And satisfaction with the care, represented by the nurses' interest in women's health, the disposition to help, dialogue, clearing doubts, care for pain, emotional support, promoting the mother's selfconfidense^55^ ^,^ ^56^ ^.^

Breastfeeding, as one of the most preponderant factors in the woman's adequate adaptation to postpartum, merits our keeping nursing care in mind. In qualitative synthesis conducted in Western nations, from 1990 to 2007, on support to mothers during breastfeeding, it was found that in spite of increasing their knowledge, they continue manifesting their lack of satisfaction regarding their experiences with such practice. The analysis of the study reports that mothers tend to look for more social support than for healthcare services, since these are described as unfavorable, due to time constraints, lack of availability of healthcare professionals, promotion of useless practices, and contradictory advise^57^.

Postnatal care offered by nursing, including health education, must respond to the individual needs of women and their families, permitting them the construction of their own learning, based on prior experiences; care should focus on ''being with'' the puerperium woman when she requires real nursing care.

## Conclusions

The review conducted suggests the need to show the effectiveness of the nursing interventions during the postnatal stage to favor the coping and adaptation process of mothers during puerperium. Available literature on the subject reveals the following:

The evaluation of the four adaptation modes - the physiological, the self-concept, the role, and the interdependence - permits nursing and healthcare professionals to view puerperium women in comprehensive manner; that is, care must tend to the physiological needs of the person, bearing in mind what she thinks of herself, the functions she performs in society, and her interaction with the rest.

It is necessary to promote self-care by teaching women to differentiate between the changes involved during puerperium and the real signs of alarm. In turn, nursing should motivate women to analyze the roles they must assume during postpartum and their posture before them.

In interdependence, we must bear in mind the socio-cultural aspects surrounding maternity; myths and beliefs come about with which nurses must maintain, negotiate, or restructure the care actions. For many cultures, puerperium is a transition stage, where the woman's perception of her external and internal world changes.

Nursing can promote the expression of emotions around maternity, including prior family experiences, given that - frequently - women reconstruct the maternal model in their children.

Everyday problems of postpartum, like tiredness, stress, sexual feelings, pain, breast problems, and lack of satisfaction with the physical appearance must also be considered to improve the coping capacity. Not caring for these types of problems may lead to altering the family dynamics and even to postpartum depression.

Literature reflects three recourses of support that influence in coping and adaptation during puerperium: The first is the family, especially the partner, the mother of the puerperium woman, or any of the women in the family group, given that upon the difficult situations of puerperium, the other family members become support recourses that propose coping strategies and contribute to decision making.

The second recourse is education, which has traditionally centered on gestation and partum, but little on the real needs of women and their families during puerperium. This is influenced by the novelty of gestation, with nursing being responsible for alerting on problems of postpartum adaptation and intervening upon the real necessities manifested by women during puerperium.

The third recourse refers to current norms and the role of nursing professionals during puerperium. Although national and international legislation is inclined toward improving maternal and perinatal healthcare, healthcare professionals still lack awareness on the importance of this period and its relevance on the mental and physical health of the woman, the child, and the family.

Nursing must recognize that although women with prenatal education may have a higher level of knowledge on themes like breast-feeding and on some self-care practices, many women manifest that this is not sufficient when they encounter problems regarding puerperium, making it necessary to have additional nursing interventions during this stage, like direct accompaniment for the puerperium woman and her family, with strategies like home visits and telephone monitoring.

The authors thank Universidad de La Sabana for the support offered for this publication. We, herein, declare having no conflict of de interest with the institution sponsoring the research or where the research was conducted. 
